# Endothelin-1 Serum Concentration is Lower in Children and Adolescents with High Myopia, a Preliminary Study

**DOI:** 10.3390/jcm9051327

**Published:** 2020-05-02

**Authors:** Katarzyna Powierza, Beata Żelazowska-Rutkowska, Jolanta Sawicka-Powierza, Bożena Mikołuć, Beata Urban, Wojciech Zaremba, Bogdan Cylwik, Alina Bakunowicz-Łazarczyk

**Affiliations:** 1 University Clinical Hospital in Bialystok, Medical University of Bialystok, 15-089 Białystok, Poland; kpowierza93@gmail.com; 2 Department of Pediatric Laboratory Diagnostics, Medical University of Bialystok, 15-089 Białystok, Poland; beata.zelazowska@umb.edu.pl (B.Ż.-R.); cylwikb@umb.edu.pl (B.C.); 3 Department of Family Medicine, Medical University of Bialystok, 15-089 Białystok, Poland; jolasawicka@gmail.com; 4 Department of Pediatrics, Rheumatology, Immunology and Metabolic Bone Diseases, Medical University of Bialystok, 15-089 Białystok, Poland; bozenam@mp.pl; 5 Department of Pediatric Ophthalmology and Strabismus, Medical University of Bialystok, 15-089 Białystok, Poland; urbanbea@umb.edu.pl (B.U.); wojciech.zaremba73@gmail.com (W.Z.)

**Keywords:** endothelin-1, high myopia, axial length of eye, children, adolescents

## Abstract

The aim of this study is to evaluate the levels of enothelin-1 (ET-1) in children and adolescents with high myopia and its association with the axial length of the eye and the presence of myopic retinal degeneration. The cross-sectional study was carried out in 57 patients with high myopia and 29 control subjects. Serum concentrations of ET-1 were measured using enzyme-linked immunosorbent assay (ELISA) kit. A significantly lower concentration of ET-1 in highly myopic patients compared to controls was found (1.47 (0.91; 1.87) vs. 1.94 (1.1; 2.69) pg/mL, *p* = 0.005). In patients with high myopia, a weak negative correlation between ET-1 concentration and the longest axial length out of the two eyes was found (*r* = −0.255, *p* = 0.0558). Further analysis revealed statistically significant differences in ET-1 concentration between patients with the axial length of the eye > 26 and ≤ 26 mm (*p* < 0.041) and patients with the axial length of the eye > 26 mm and controls (*p* < 0.001). ET-1 expression is disturbed in highly myopic children and adolescents. Lower ET-1 concentration in patients with the axial length of the eye > 26 mm may co-occur with high myopia and should be considered a risk factor in the pathophysiology of high myopia progression.

## 1. Introduction

Myopia is one of the most common eye disorders in the world and its prevalence remains higher in Asia (60%) compared with Europe (40%). Studies report high prevalence rates in schoolchildren in East Asia (73%) and North America (42%). Low prevalence below 10% has been demonstrated in African and South American children. Risk factors for myopia in schoolchildren include limited outdoor time, dim light exposure, use of LED lamps for homework, insufficient amount of sleep, reading distance less than 25 cm, and living in an urban environment [1]. Pathological or high myopia is usually defined as spherical equivalent (SE) ≥ −6.00 diopters (D) or the axial length of the eye > 26 mm. This type of myopia is a public health and economic challenge because of its association with potentially blinding complications such as glaucoma, retinal detachment, myopic macular degeneration, and ultimately blindness [2,3,4]. In recent years the occurrence of high myopia has also increased at an alarming rate in adults and elevated intraocular pressure is a risk factor for high myopia [5].

Endothelin-1 (ET-1) belongs to the family of endogenous vasoconstrictor peptides [6]. ET-1 is widely distributed in human tissues and secreted mainly by vascular smooth muscle cells (VSMC). As a result of transcription, proendothelin-1 is formed. Subsequent stimulation by hypoxia or vascular wall shear stress with the participation of an endothelin-converting enzyme leads to the formation of an active peptide [7]. ET-1 exerts its effects by binding to the endothelin receptors A (ETA) and B (ETB), two cell surface proteins that belong to the G-protein-coupled receptors superfamily. Both these receptors induce various effects depending on the binding subtype of endothelin. They also differ in their affinity to each type of endothelin. The binding affinity of ET-1 to ETA is greater than that of ET-2 and ET-3, whereas all subtypes of endothelin have equal binding affinity to receptor ETB [8]. ETA receptors are located mostly in VSMC, where they are responsible for potent vasoconstriction, cell proliferation, and a proinflammatory effect. ETB receptors include two subtypes: ETB1, which is expressed on endothelial cells and results in nitric oxide-mediated vasodilation, and ETB2, present in VSMC, which causes vasoconstriction [9,10].

Since we treat a great number of young patients with progressive myopia in our outpatient clinic, the pathogenesis of myopia is our object of interest. In our previous studies we have evaluated serum and hair concentrations of antioxidant microelements—zinc, copper, selenium, manganese, and Cu/Zn ratio in children and adolescents with myopia [11,12]. We have observed low serum concentrations of zinc and selenium in myopic children, which may imply an association between deficiency of these antioxidant microelements and the development of myopia, and additionally, significantly higher Cu/Zn ratio, which can suggest a relationship between myopia and oxidative stress. There are numerous literature reports on the involvement of environmental and genetic factors in the development of myopia, but there is limited data regarding cellular mechanisms. Up to date, researchers have not been successful in identifying a molecule common to all metabolic pathways which could be a target for effective treatment against myopic progression. Taking into account the physiological importance of ET-1 level in blood flow, we hypothesized that circulating ET-1 levels may vary depending on the severity of high myopia. Therefore, we determined ET-1 concentration in high myopia children and adolescents and examined its association with the axial length of the eye and the presence of myopic retinal degeneration.

## 2. Materials and Methods

### 2.1. Participants

The cross-sectional study was conducted among children and adolescents with high myopia and healthy controls who were recruited from the Department of Pediatric Ophthalmology and Strabismus, Medical University of Bialystok, Poland. Exclusion criteria were any systemic inflammatory disease, arterial hypertension, diabetes mellitus, kidney diseases, hyperthyroidism, any anterior or posterior eye segment disease, or any systemic medication (e.g., steroids, nonsteroidal anti-inflammatory drugs, vitamins). Sixty-five patients with high myopia and thirty-four healthy subjects were recruited into the study. Eight individuals with high myopia as well as five controls did not meet the eligibility criteria and were excluded. Informed consent was obtained from all subjects or their legal guardians.

Finally, 57 Caucasian individuals (31 females, 26 males) aged 7–17 years with high myopia, defined as SE ≥ −6.0 D and 29 (17 females, 12 males) age- and gender-matched healthy subjects from 7 to 17 years were recruited into the study. All control individuals had full visual acuity and did not require correction.

The study was performed in accordance with the Declaration of Helsinki on Biomedical Research Involving Human Subjects. The study protocol and procedures were approved by the Ethics Committee of the Medical University of Bialystok (No R-I-002/144/2019).

### 2.2. Measurements

After the participants had rested in the sitting position for 30 min, blood samples were collected after overnight fasting from peripheral veins. The serum was separated by centrifugation immediately at 1.500× *g* for 10 min at room temperature and sera were collected and stored at −80 °C until measurements were obtained.

The concentration of ET-1 in serum was determined by sandwich enzyme-linked immunosorbent assay (ELISA) using a commercially available kit Endothelin-1 Immunoassay (R@D Systems, Inc., Minneapolis, MN, USA). ELISA kit was used following the manufacturer’s instructions. In this technique a monoclonal antibody specific for ET-1 was pre-coated onto the wells of a 96-well microplates provided in this set. Next, standards and patient samples were pipetted into the wells and any endothelin-1 present was bounded by the immobilized antibody. After washing away any unbound substances, an enzyme-linked monoclonal antibody specific for ET-1 was added to the wells. Following a wash to remove any unbound-enzyme reagent a substrate solution was added to the wells. A substrate solution was acted upon by the enzyme to produce color in proportion to the amount of ET-1 bound in the initial step. The intensity of this colored product was directly proportional to the concentration of ET-1 in the patient specimens. Readings were performed using an ELISA microplate reader (ANTHOS, Wals/Salzburg, Austria) at an absorbance value of 450 nm. The results were calculated based on the standard curve and expressed in pg/mL. Sensitivity of the assay was 0.207 pg/mL. The coefficients of variation values (CV%) of intra-assay precision was 2.3% for 7.34 pg/mL and 1.9% for 14.7 pg/mL and inter-assay precision was 5.9% for 4.43 pg/mL and 5.3% for 14.4 pg/mL.

All patients underwent detailed ophthalmologic examination including visual acuity measurement, refraction error examination, intraocular pressure with TonoPen, slit-lamp examination and dilated fundus examination using Volk lens. Refractive error was determined using cycloplegic refraction after application of 1% Tropicamide with a TONOREF™ Nidek’s autorefractor keratometer pachymeter. All measurements of an axial length of the eye were obtained using ultrasound A scan Quantel Medical AVISO device.

### 2.3. Statistical Methods

The statistical analysis was conducted using the STATISTICA version 13 [13]. Qualitative characteristics (sex, age group, presence of myopic retinal degeneration) were presented as numbers and percentages. Quantitative parameters were presented as mean, standard deviation, range, median, and quartiles. The structure by sex and age subgroups were compared using the Pearson chi-square test. Mann-Whitney U test was applied for comparison of analyzed features between all subgroups. Correlations between parameters were evaluated with Spearman’s rank correlation coefficient. A significance of the coefficient was assessed by the t-student test. Differences in the levels of analyzed parameters and correlations were considered statistically significant at *p* < 0.05.

## 3. Results

The basic characteristics of high myopia patients and controls, as well as median serum ET-1 concentrations, are summarized in Table 1. As shown below, statistically significantly lower concentration of ET-1 in patients with high myopia compared to controls was demonstrated. No correlation was established between ET-1 concentration and age, either in highly myopic patients or controls (*r* = 0.124, *p* = 0.364 or *r* = 0.069, *p* = 0.772, respectively).

Characteristics of clinical parameters of patients with high myopia are presented in Table 2. All patients had normal intraocular pressure of 14–18 mmHg. None of the myopic patients had peripheral retinal degeneration (e.g., lattice degeneration). Advanced peripheral chorioretinal atrophy was observed in 11 patients with refractive errors between −8.0 to −17.0 D. In the remaining patients peripheral chorioretinal atrophy was less pronounced. In patients with high myopia, a weak negative correlation between ET-1 concentration and the longest axial length out of the two eyes was found (*r* = −0.255, *p* = 0.0558). The axial length of the eye was negatively correlated with SE, both the right and left eyes (*r* = −0.428, *p* < 0.001; *r* = −0.483, *p* < 0.001, respectively). A positive correlation between SE refractive errors of both eyes was noticed (*r* = 0.63, *p* < 0.001).

**Figure 1:**
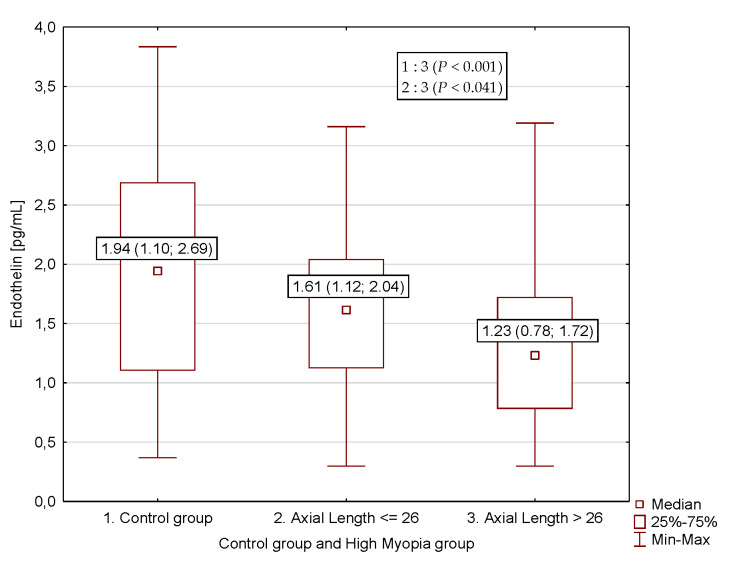
Endothelin-1 concentrations in myopic patients with the axial length of the eye > 26 and ≤ 26 mm and controls.

In further analyses the relationship between ET-1 concentrations and patient features were assessed in subgroups, taking into account: gender, age (≤13/>13 years of age), axial length of the eye (> 26 and ≤ 26 mm) and presence of severe peripheral chorioretinal atrophy. Based on these analyses, no significant differences were found between ET-1 levels in different patient subgroups when gender (*p* = 0.841), age (*p* = 0.942), and peripheral chorioretinal atrophy (*p* = 0.649) were taken into consideration. Significantly lower ET-1 concentration was found between patients with the axial length of the eye > 26 and ≤ 26 mm (*p* < 0.041) and between patients with the axial length of the eye > 26 mm and controls (*p* < 0.001). There was no difference between ET-1 concentration in myopia patients with the axial length of the eye ≤ 26 mm and controls. No significant differences were found between subgroups of myopic patient with the axial length of the eye > 26 mm, with the axial length of the eye ≤ 26 mm and controls, when gender (*p* = 0.312) and age (≤13/>13 years of age) (*p* = 0.912) were taken into consideration (Table 3, Figure 1).

In subgroups of patients, taking into account the axial length of the eye ≤ 26 and > 26 mm, statistically significant differences between means of refractive errors of right and left eyes were observed [−6.9 ± 1.7 (−11–−4.3) vs. −8.5 ± 3.2 (−18–−3); *p* = 0.039 and −6.7 ± 1.6 (−10.5–−3.5) vs. −9 ± 3.8 (−20–−3.8) D, *p* = 0.014, respectively].

## 4. Discussion

In the current study we concluded that endothelin-1 (ET-1) expression is disturbed in children and adolescents with high myopia. The significantly lower concentration of ET-1 observed in patients with the axial length of the eye > 26 mm, both male and female, in comparison to myopia patients with the axial length of the eye ≤ 26 mm and to the controls may indicate disturbance in the endothelin signaling pathway and its role in the pathophysiology of high-myopia eyes. A weak negative correlation between ET-1 concentrations and the longest axial length out of the two eyes, and a strong negative correlation between the axial length of the eye and SE refractive error in both eyes suggest an association between lower ET-1 concentrations and severity of high myopia. Low serum ET-1 levels in our study may co-occur with high myopia and may be due to insufficient expression or rapid elimination processes of ET-1. Circulating ET-1 is mainly eliminated in the lung. The primary mechanism of elimination in the lungs and liver is by the internalization of the ETB (ETA) receptor and ET-1 complex, whereas in the kidneys by degradation by neutral endopeptidase (probably the most active in the proximal tubules) [14]. To the best of our knowledge, our study is the first to demonstrate a lower level of ET-1 in patients with high myopia and its relationship with the axial length of the eye, and for this reason we cannot compare our results with those of other authors.

Myopia is the most common eye disorder in children and adolescents. It is a condition in which individuals can see close objects clearly, while objects that are farther away appear blurred. In most cases, this condition occurs when the eyeball is too elongated, relative to the focusing power of the cornea and lens of the eye. This causes the light rays to focus at a point in front of the retina, not directly on its surface. In some cases, the eyeball is much more elongated, and this condition is called high or pathological myopia. Less commonly, myopia can also be caused by the cornea and/or lens being too curved for the length of the eyeball. In some cases, myopia occurs because of a combination of these factors. Myopia is a complex disease with a multi-factorial etiology. Many experimental and clinical studies have shown that myopia development results from the interplay between genetic and environmental factors [15,16]. Numerous studies demonstrate a higher rate of myopia in children with myopic parents suggesting that genetic factors are clearly involved in myopia development. At the same time, population studies indicate that development of myopia is associated with education, time spent outdoors, and the reading distance of less than 25 cm.

There is increasing evidence that the endothelin signaling pathway plays a key role in the pathogenesis of several ocular diseases and ET-1 level is a useful marker for evaluation of intraocular hemodynamic. The amount of blood needed in various organs and tissues varies and is achieved by adapting perfusion pressure and local resistance, for which the diameter of vessels is responsible [17]. In the eyeball, endothelins are expressed in tissues such as corneal epithelium, optic nerve astrocytes, ciliary body, trabecular meshwork cells, as well as in the vascular endothelial cells in the choroid and retina [18]. ET-1 modulates pericyte contractility in order to regulate retinal blood flow [19] and is involved in the regulation of intraocular pressure and aqueous humor dynamics [20]. Factors known to promote ET-1 expression include thrombin, insulin, cyclosporine, epinephrine, angiotensin II, cortisol, inflammatory mediators, hypoxia, and vascular wall shear stress. Endothelin expression is inhibited by nitric oxide (NO), dilator proteinoids, and natriuretic peptides [21]. The concentration of ET-1 in plasma depends on several factors such as synthesis, receptor connection, and its elimination from the body (lung, kidney, and liver) [22].

Reduced saturation of arterioles in the retina as well as narrowing of the retinal vascular diameter in highly myopic eyes has been previously demonstrated [23,24,25,26,27]. Many researchers have also consistently reported lower blood flow parameters in myopic eyes, regardless of the diagnostic methods used [28]. As a result of axial eyeball growth in patients with high myopia, laminar blood flow as well as retinal vascular tone are disturbed. Consequently, it affects the vascular shear stress, which is necessary to form the active ET-1 peptide [7], responsible for vasoconstriction. At a later stage, ET-1 releases NO leading to permanent tonic vasodilation [29]. Inhibition of basal NO release in humans results in vascular smooth muscle hypertrophy. The physiological value of shear stress in the arteries is in the range of approximately 15 dyne/cm^2^ to 70 dyne/cm^2^. Such shear stress exerts a protective effect on the vascular endothelium by stimulating the production of vasodilators. The thickening of the inner membrane occurs in places where wall shear stress is less than 10 dyne/cm^2^. Experimental studies using flow chambers have shown that the endothelium already responds to very small changes in the shear stress value [30]. All this information, as well as reduced ET-1 expression in our highly myopia patients indicates disturbance of the endothelin signaling pathway, which is necessary for proper functioning of the vascular endothelium.

The two major limitations of our study were the small sample size and the observational design of the study. This did not allow us to draw firm conclusions about causality. In addition, the inability to assess the concentration of factors known to promote the expression and inhibitors of ET-1 in this study may limit the spectrum of possible associations.

In spite of the limitations of the study, we found that the expression of endothelin-1 is disturbed in high myopia pediatric patients with the axial length of the eye > 26 mm, and the lower level of ET-1 may co-occur with high myopia and may play a role in chorioretinal circulation and in the pathophysiology of high myopia progression. Therefore, the assessment of ET-1 levels in patients with high myopia may provide additional insights into choroidal and retinal hemodynamic and to the development of new therapeutic strategies to prevent myopic blindness. However, further studies involving a larger number of participants and longer follow-up duration are required to confirm our results.

## Figures and Tables

**Table 1:** Basic characteristics and serum endothelin-1 concentration in children and adolescents with high myopia and controls.

Parameter	High Myopia Patients	Control Group	*p* Values
Number, *n*	57	29	
Age, years	14 (13; 16)	14 (11; 15)	0.181
Gender (female/male), *n* (%)	31 (54)/26 (46)	17 (59)/12 (41)	0.709
Age group (≤13/>13 year), *n* (%)	23 (40)/34 (60)	13 (45)/16 (55)	0.691
Endothelin-1, pg/mL	1.47 (0.91; 1.87)	1.94 (1.1; 2.69)	0.005

Notes: The results are presented as medians and quartiles (Q1; Q3), or numbers (*n*) and percentages (%).

**Table 2:** Characteristics of clinical parameters of patients with high myopia.

Features	High Myopia Patients, *n* = 57
Visual acuity of right eye	1.0 (0.7; 1.0)
Visual acuity of left eye	0.8 (0.6; 1.0)
Refractive error of right eye, D	−6.8 (−8.6; −6)
Refractive error of left eye, D	−6.8 (−9.5; −6)
Axial length of right eye, mm	25.9 (25.3; 26.6)
Axial length of left eye, mm	25.8 (25.3; 26.6)
Peripheral chorioretinal atrophy, yes/no, *n* (%)	11 (19)/46 (81)

Notes: The results are presented as medians and quartiles (Q1; Q3), or numbers (*n*) and percentages (%). Abbreviations: D, diopter; mm, millimeter.

**Table 3:** Concentration of endothelin-1 in children and adolescents with high myopia.

Parameter	High Myopia Patients		Control Group
	Axial Length of Eye		
	>26 mm	≤26 mm	
Number, *n*	29	28	29
Gender (female/male), *n* (%)	13 (45)/16 (55)	18 (64)/10 (36)	17 (59)/12 (41)
Age group (≤13/>13 year), *n* (%)	12 (41)/17 (59)	11 (39)/17 (61)	13 (45)/16 (55)
Endothelin-1, pg/mL	1.23 (0.78; 1.72) ^a,b^	1.61 (1.12; 2.04)	1.94 (1.1; 2.69)

Notes: The longest axial length out of the two eyes was enrolled. The results are presented as medians and quartiles (Q1; Q3). ^a^ *p* < 0.001—between patients with the axial length of the eye > 26 mm and controls; ^b^ *p* < 0.041—between patients with the axial length of the eye > 26 and ≤ 26 mm.
